# Preface

**DOI:** 10.1093/jac/dkab362

**Published:** 2021-11-21

**Authors:** John D Perry, Russell Hope, Russell Lewis

This Supplement offers four articles addressing current challenges in the management of serious bacterial infections. In the opening article, Mark Wilcox and Matthew Dryden provide an overview of the epidemiology of healthcare-acquired bacterial infections in acute and long-term care facilities. Particular focus is given to assessing the healthcare burden of complicated skin and soft tissue infections (SSTIs) including surgical site infections. The epidemiology and general management of SSTIs is discussed and sets the scene for Christian Eckmann and Paul Tulkens, who review treatment options for SSTIs including a detailed assessment of delafloxacin and the long-acting lipoglycopeptide agents, dalbavancin and oritavancin. Valuable insights are provided into the consequences of under- and over-treatment, economic considerations, and the goal of combining effective treatment with optimal antimicrobial stewardship.

The remainder of this Supplement offers a timely review of treatment strategies for multidrug-resistant Gram-negative bacteria. Matteo Bassetti and Javier Garau assess the burden of such infections and outline treatment options for a range of infection types. They discuss in detail the efficacy of old and new antimicrobials and offer a glimpse into the antibiotic pipeline to assess the potential of future treatments. The importance of local epidemiology and rapid diagnostics is also considered. Emilio Bouza continues this theme and focuses on carbapenems combined with β-lactamase inhibitors. An in-depth assessment of meropenem/vaborbactam is provided that covers pre-clinical and clinical trials as well as ‘real world’ outcomes for clinically challenging cases and/or extensively drug-resistant bacteria.

The Supplement provides a valuable overview of the increasingly complex antibiotic-resistant bacteria in circulation and therapeutic options for the common infections they cause.

